# Supplementary figures and images for: Self-selection of food ingredients and agricultural by-products by the house cricket, Acheta domesticus (Orthoptera: Gryllidae): A holistic approach to develop optimized diets
Source: PLoS One. 2020 Jan 24;15(1):e0227400. doi: 10.1371/journal.pone.0227400 (PMC6980616; doi:10.1371/journal.pone.0227400)

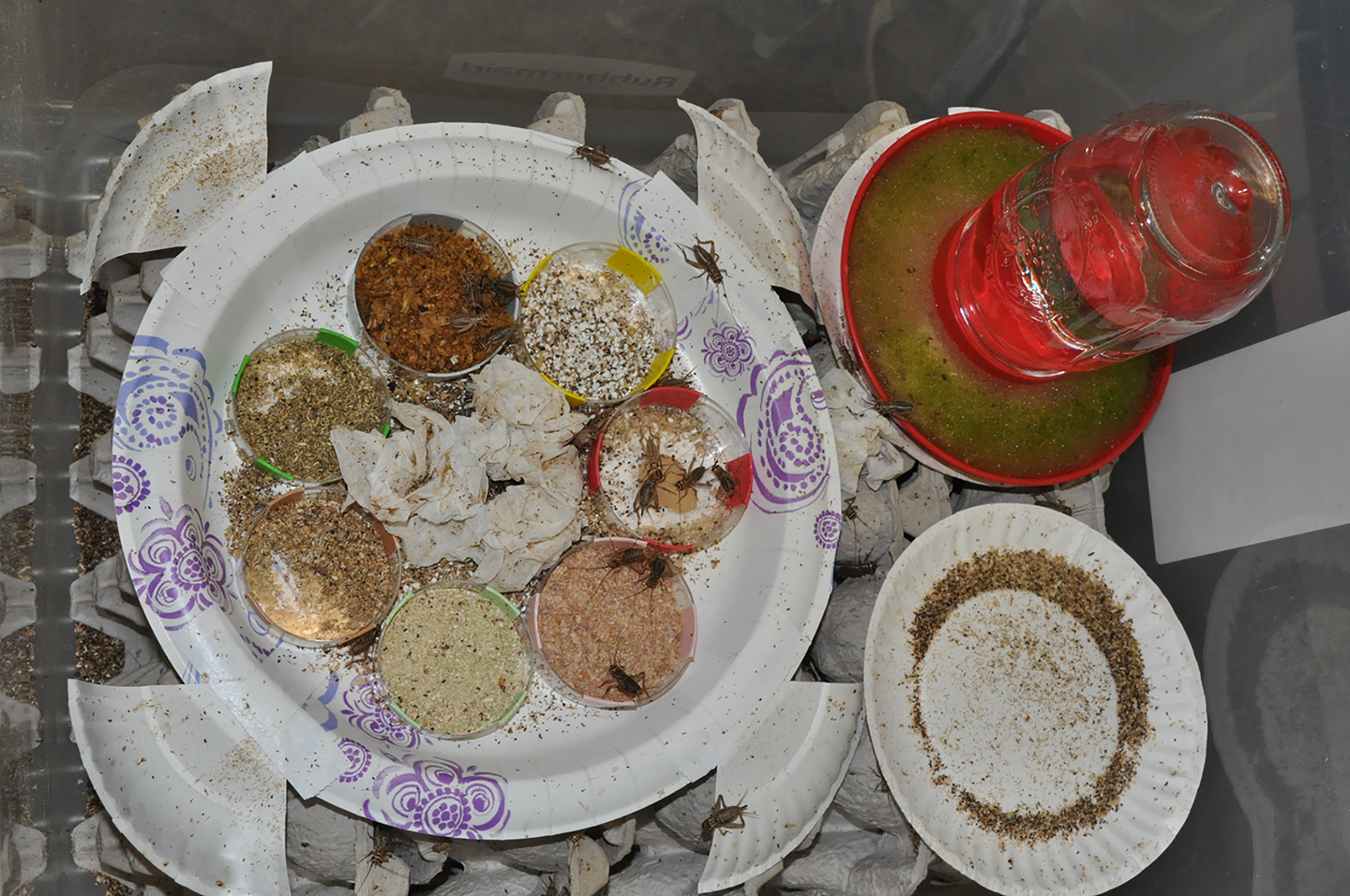

Supplement: S1 Fig — In this example from treatment BP1, the water dispenser (right up corner) and the radially-distributed food choices (left centre) are sitting on top of the egg cartons (rearing substrate), Color-coded food choices from the top in clockwise direction include: corn DDGS (grey), buckwheat seed (yellow), brewer’s yeast (red), wheat bran (pink), cabbage dry (light green), peanut hulls (brown), and alfalfa pellets (green). (TIF) [file pone.0227400.s001.tif]
