# Supplementary material for: Self-selection of food ingredients and agricultural by-products by the house cricket, Acheta domesticus (Orthoptera: Gryllidae): A holistic approach to develop optimized diets
Source: PLoS One. 2020 Jan 24;15(1):e0227400. doi: 10.1371/journal.pone.0227400 (PMC6980616; doi:10.1371/journal.pone.0227400)
Supplement: S1 Text — (DOCX) [file pone.0227400.s002.docx]

Supplemental Material S1 Text: Production of by-products in the United States

In the United States the biofuel industry produces high amounts of by-products resulting from ethanol and biodiesel production [1]. The main by-product of ethanol production in the U.S. is dry distillers’ grains or DDG’s. In the U.S. DDG’s are mostly from corn and wheat origin and marketed as DDGS (dry distiller’s grain with solubles) [2]. An estimated 90,000 tons of DDGS are produced weekly in the U.S, but only a small portion is sold locally as animal feed [3]. Biodiesel is mostly produced from vegetable oil in the U.S. and the principal source is soybean oil [1]. The oil is extracted using solvents for a more efficient recovery leaving defatted soybean meal as a by-product unsuitable for human consumption. This by-product is high in protein with a minimum content of 47.5% protein and 3.3% fiber [4]. Canola meal is a by-product of canola oil production and high amounts are produced yearly. Canada produced 4 million tonnes of canola meal in 2013 and 2014 [5] and the U.S. produced an estimated 1 million tonnes in 2016 [6]. Rice bran is another important agricultural by-product. The world production of rice was 631 million tonnes in 2006. The U. S. produced 1.5% with an estimated 9.5 million tonnes during this year [7]. Rice bran constitute 12% of the whole grain rice weight and annually 63 to 76 million tonnes are produced in the world. Rice bran has a high oil content, which degrades quickly due to enzymatic action and requires a high temperature treatment to stabilize it [7]. The rice bran oil has a high content of polyunsaturated fatty acids (35.6%) and phytosterols (1.8%), which makes it a valuable product for the health foods market. Approximately 15% to 20% of the rice bran produced in the U.S. is processed for oil extraction leaving defatted rice bran as a secondary by-product [7].

References:

1. US Department of Agriculture (USDA). U.S. Bioenergy statistics. U. S. Department of Agriculture, Economic Research Service; 2019a. <https://www.ers.usda.gov/data-products/us-bioenergy-statistics/>
2. U. S. Grains Council. A guide to distiller’s dried grains with solubles (DDGS). 3rd ed. Washington (DC): U. S. Grains Council; 2012.
3. Iowa Corn. Distillers grains. Iowa Corn Promotion Board / Iowa Growers Association; 2019. <https://www.iowacorn.org/corn-uses/livestock/distillers-grains/>
4. Blasi DA, Drouillard J, Titgemeyer EC, Paisley SI, Brouk MJ. Soybean hulls. Kansas Agricultural Experiment Station, contribution No. 00-79-E, Manhattan (KS): Kansas State University; 2000.
5. Canola Council of Canada (CCC). Canola meal feeding guide. Winnipeg (Manitoba): Canola council of Canada; 2015.
6. U.S. Canola Association. Crop production: seed, meal and oil; 2019. <http://www.uscanola.com/site/epage/102391_956.htm>
7. Kahlon, TS. Rice bran: production, composition, functionality and food applications, physiological benefits. In: Cho SS, Samuel P, editors. Fiber ingredients: Food applications and health benefits. Boca Raton (FL): CRC Press, Taylor & Francis Group; 2009. p.305-321.
