# Supplementary material for: Self-selection of food ingredients and agricultural by-products by the house cricket, Acheta domesticus (Orthoptera: Gryllidae): A holistic approach to develop optimized diets
Source: PLoS One. 2020 Jan 24;15(1):e0227400. doi: 10.1371/journal.pone.0227400 (PMC6980616; doi:10.1371/journal.pone.0227400)
Supplement: Attachment — Submitted filename: PONE-D-19-2244R1-Answer to reviewers.docx [file pone.0227400.s008.docx]

Answer to reviewers:

**Editor Comments:**

Thank you for submitting your manuscript to PLOS ONE. After careful consideration, we feel that it has merit but does not fully meet PLOS ONE’s publication criteria as it currently stands. Therefore, we invite you to submit a revised version of the manuscript that addresses the points raised during the review process.

The current version of the manuscript has much improved, however there are some details that still need to be addressed. Please carefully consider the reviewer's comments and recommendations.

Additional Editor Comments (if provided):

I agree with the reviewer that Fig. 2 should go into the Supplementary material (By the way, re: reviewer´s comment #4, on Fig. 2 I see the egg-cartons placed horizontally, not vertically).

Figure 2 has been moved to supplementary materials and all the rest of the figures have been renumbered.

Indeed, the stack of cartons were placed horizontally. Reviewer 1 may have been thinking about individual cartoon pieces on the whole stack.

Line 129 - ...for a diet that convergeS (an 's' is missing)

Corrected.

Line 445-446: I find the phrasing '..using THE generalized linear mixed model' odd, since there is not only one GLMM (i.e., you should say 'a' GLMM). I also agree with the reviewer that the Methods would be much clearer if you could organize better the variables and models you ran (e.g., Model 1: response variable xx, random factor xx, fixed factors xx; Model 2: xxx, etc...)

The description of GLMM has been place between parentheses instead of GLMM.

Description of the models have been added to the text.

Line 453: you still mention 'mixed effects GLM'

Corrected.

Reviewer #1: Thank you to the authors for their revision of manuscript PONE-D-19-22442R1.

1. I must say it was a bit difficult to assess the revision of this manuscript because in their response to reviewers’ letter, authors did not indicate the line number of the changes in the manuscript. Please always include line numbers in your response to reviewers’ letter. Otherwise, you make the work of reviewers unnecessarily difficult. There are also inconsistencies between the response to reviewers’ letter and the actions taken in the manuscript. For instance, response to Specific comment # 13 reads "... We respectfully decline to add pricing of products in the introduction"; however, prices were actually included in the introduction (L 72-73). This situation made me feel confused.

We did not indicate the line numbers where changes were done because we were under the impression that reviewers had access to the “track changes” version of the manuscript. We will provide line numbers this time. We apologize for the inconsistencies, but clarity is also needed from the reviewer’s side. Some of the comments were difficult to understand and we were just trying to address them the best we could. We will try to do better this time.

2. The manuscript still has sections that should improve in precision and clarity. Most of them related to the experimental design and statistical analyses. In this regard, I think authors misunderstood my comment # 8, as I did not say it was "necessary" to include a graphical representation of the experimental designs. What I say, was that "readers would be greatly benefited" if such a figure was included. I have no objection if authors refuse to follow this comment. However, consider that many readers will find it much easier to understand complex studies with a graphical explanation. But again, this is not mandatory, but rather something that could add to improve the comprehension of the study.

The way we interpreted some of the comments from reviewer 1 may have not been as intended. We are also confused by difficulties of the reviewer to understand the statistical procedures, which are not outside of the standard for this type of studies. Reviewer 2 from the first submission, did not have such difficulties.

3. What I must insist, is that the response and explanatory variables considered in experiments and analyses must be clearly mentioned in the materials and methods section. For instance, it is not completely clear how the "Data consisting of ending biomass and total food consumed" (L 279) was measured and its units. Also, in the results section it is mentioned that "Sex had a significant impact on adult dry weight..." (L 667-668), but in the materials and methods section it is never mentioned that sex was considered as an explanatory variable in the models fitted to data. Authors must link all the pieces of information in the materials & methods with the results to improve the flow of ideas and the comprehension of their study.

A sentence was missing explaining that crickets were weighed at the end of the experiment. This was added (Lines 254-256).

How food consumption was calculated is well explained in the first paragraph in “Data Analysis”. Explanation of how the data was collected is explained in the previous paragraph before the section “Data Analysis” (lines 258-264).

Please see these: “After six weeks, experimental units were monitored daily for the presence of adult crickets. When present, adult **crickets were sexed**, weighed, and frozen at -25ºC. The live weight and **sex of each cricket** was recorded along with its corresponding experimental unit number, diet treatment and date of emergence.” Lines (426-429).

4. Throughout the materials and methods section, it is mentioned that egg cartons were placed horizontally, but in the figure 2 it seems to me that the cartons were placed in vertical position.

Horizontally is correct

5. Correct kilogram symbol throughout the manuscript (kg - correct; Kg - incorrect).

This has been corrected throughout the text.

6. L 42, the remaining 38 and 8% of new diet formulations consisted of...

This is incorrect. The percentages as stated in the text are correct.

7. L 67-68, "... primitive rearing practices that require..." this is not necessarily true. There are companies using state-of-the-art technologies to rear crickets. For instance, see ASPIRE food group.

Some companies, unknowingly to our team, may have been achieving progress in this area, but they are a very small exception of the commercial companies world-wide. Still, we submit that “state of the art” may still be primitive as compared to livestock industry.

8. L 92-94, what nutrients, exactly, are found in by-products, and cite reference.

References have been provided and (lines 206-207). Enumerating the nutrients of each by-product will require additional tables, but this is unnecessary because the literature provided has this information in downloadable form.

9. L 151, indicate conditions of egg hatch.

Done (Line 152).

10. L 153, indicate long, wide and tall. Applies to all instances in which size is indicated.

Done (Lines 153, 166, 171, 181, and 407).

11. L 199-202, is there another way in which authors can present this information? In its

current form it is confusing.

This way is no confusing is just a simple mathematical operation.

12. In Table 1, please include the amount of each food that was offered to crickets.

Done.

13. L 232-236, I think this figure indicate proportions not ratios.

Ratios is correct, but they are in three axes instead of two.

14. In Table 2, food ingredients of diet 2 do not sum to 100; please correct. Suggest

"Estimated nutrient proportions" instead of "Estimated nutrient ratios".

Corrected (Thank you for this one).

15. L 333-334, indicate how much food was added to Petri dishes.

Done (Lines 328-330).

16. L 339, remove extraneous period.

Done (thank you).

17. L 381-382, remove "The live weight...", this is already mentioned at the end of last

paragraph.

Corrected (Lines 376-377).

18. L 428-429, how much food was provided to crickets.

This information was added (Line 415).

19. Line 505 - Table 3, indicate the period in which these foods were consumed, and the number of crickets that consumed this amount of food.

This is well explained in “Materials and Methods” (Lines 179-180 and 239-243). Experiment duration and initial cricket population per experimental unit. Crickets were not counted at the end because this information was no relevant for the self-selection study. Relative ingredient consumption was the relevant information required.

20. I am wondering if some of the tables or figures (perhaps figure 2) could be moved to

supplementary material.

To be able to satisfy this, we need to know specifically what tables and an explanation of why it would be better to move them to supplementary material. The tables are currently presenting information that it would be difficult to present in any other way.
